# Supplementary material for: Information transfer efficiency differs in wild chimpanzees and bonobos, but not social cognition
Source: Proc Biol Sci. 2020 Jun 24;287(1929):20200523. doi: 10.1098/rspb.2020.0523 (PMC7329035; doi:10.1098/rspb.2020.0523)
Supplement: Supplementary Information [file rspb20200523supp6.pdf]

Supplementary Information for

Information transfer efficiency differs in wild chimpanzees and bonobos, but not social-cognition.

Cédric Girard-Buttoz, Martin Surbeck, Liran Samuni, Patrick Tkaczynski, Christophe Boesch, Barbara Fruth, Roman M. Wittig, Gottfried Hohmann, Catherine Crockford.

Cédric Girard-Buttoz and Roman M. Wittig
Email: cedric_girard@eva.mpg.de and wittig@eva.mpg.de

SI Methods

Study communities

The chimpanzee communities comprised 4-7 adult males, 7-14 adult females and a total of 14-25 adults and juveniles (**Table S1**). The bonobo communities comprised 5-8 adult males, 10-17 adult females and a total of 21-32 adults and juveniles (**Table S1**).

Snake model design

CGB, with the help of the IT department of the Max Plank Institute for Evolutionary Anthropology (MPI-eva), designed two 3D models of a Gaboon viper with slightly different shapes but similar proportions (Fig. S1). These two shapes were then 3D printed in two different sizes, two models being 1.15 times larger than the two other ones, to create four different models. All models were painted to mimic the color pattern of a Gaboon viper (Fig. S1). Three models were repainted during the study resulting in seven different snake models. Four of these models where used for each species with one model used for both species.

Experimental protocol

We used a three camera protocol (two handheld cameras Panasonic HC-X929 and an action camera Sony HDR-AS200 VR) to video-tape, in detail, the behavioral reactions of the apes to the snake model and their call production. CGB carried one handheld camera and filmed the individuals closest to the snake (i.e. focusing on the potential signalers). The second handheld camera was carried by the observer following the ape party and filmed the party as a whole while they approached the snake from the back focusing on the receivers. The observer filming the party from the back (i.e. farther away from the snake and the signaler than any ape present in the party) confirmed that he/she heard all alarm calls produced by commenting on the video. We assessed which individual from neighboring parties heard alarm calls using information collected by other observers following these parties on the calls they heard. Since one observer was typically following the focal party for several hours before the experiment took place, this observer had well informed knowledge of the party composition which arrived at the snake. During the focal follow, this observer recorded also party composition. For both species, a party was defined as all the individuals that an observer could see around him (i.e. within 50m). The party composition was compiled cumulatively every 30 minutes starting with all individuals that can be seen at time 0 and adding all new individuals within the following 30 minutes. Additionally, during the experiment, party composition was confirmed by the 2-4 observers present around the snake (from this project but also other projects) by checking for individual presence within 50m of the snake. In addition, the observers that were working in the forest but not in the particular party exposed to the snake also recorded any calls they heard. We assumed that all calls heard by the observer were also heard by the apes and, using combined information from several observers we obtained a more exhaustive record of which apes possibly heard which call. Before the apes arrived at the experimental location, CGB placed the wide angle

action camera, camouflaged behind leaves, 2-6m from the snake location to film the position and general behavior of as many individuals as possible. Importantly, this arrangement attempted to give simultaneous video coverage of all potential signalers and receivers within visibility. At the end of the experiment, once all the apes had moved away from the area and were out of sight, the snake model was removed.

During most experiment days, we managed to put in place the three-camera protocol (56%, 10/18, in bonobos and 65%, 15/23, in chimpanzees). For technical and practical reasons, however, we could only use two cameras in 15 experimental days (8 in chimpanzees and 7 in bonobos) and for one bonobo experimental day only one camera was used.

Video coding

Each individual entering the visual range of any video was treated as a focal. We recorded its identity, the time of arrival in the party around the snake, the time they detected the snake (if they did), whether they startled upon detecting the snake (defined as jumping or running away from the snake in a direction not in line with general travel direction), as well as the time they left the site. The instant that an individual saw the snake was clearly visible on video. The apes systematically stopped their locomotion towards the snake, looked in the direction of the snake and either startled, stared at the snake or resumed approaching the snake at a slower pace. During the entire time when the individual was visible on the video, we recorded if it was on the ground or in a tree and its distance to the snake. We coded the focal's distance to the snake (using five distance categories 0-1m, 1-5m, 5-10m and >10m), its position in space in relation to the snake model as well as its body orientation using a "compass-like" system where the snake would be in the center and the action camera in the "South" (see **Fig. S2**). The party composition around the

snake was coded continuously by combining the information about the individual present on the video with observers' comments on the video and field notes.

Mock trials

In order to reduce the habituation of the apes to the experimental situation we conducted 23 mock trials (12 in bonobos and 11 in chimpanzees) where either no snake model was used or the apes pass near but did not detect the snake model. In each mock trial the action camera was attached to a tree and the two observers filmed using the same protocol as for the experimental condition. During mock trials, only one individual chimpanzee (a juvenile male orphan) startled at the camera and produced a soft alarm call. In all other trials, no ape produced calls or expressed an apparent fear reaction to camera presence alone. We are thus confident that the behavioral reaction recorded during the experiments is related to the presence of the snake model and not a reaction to the rest of the experimental apparatus.

Habituation avoidance

We ensured that no individual saw the same snake model more than twice in different locations and, when they did, the two presentations were over a month apart in time. All bonobos and most of the chimpanzees (90%, 52/58) were subjects in four experiments or less. Five chimpanzees participated in five experiments and one chimpanzee in seven.

Inter-observer reliability

The videos of 4 experiments (2 in chimpanzees and 2 in bonobos) have been coded by a research assistant at the Max Plank institute blind to the aim of the study. Only the identities of the individuals on the videos have been provided by CGB. All inter-observer reliability on the key parameters of the analysis (i.e. timing of call production and identity of the callers, body

orientation of each individual, locomotor behavior, timing when each individual sees the snake and startle reaction) were all above 82% for each parameter for each experiment.

Definition of time slots in Model 2a and 2b

For each individual who saw the snake, its "experiment" started at the exact time it saw the snake. From that time onwards the time slot ran until the parameter "is there an ignorant individual in the audience Yes or No" changed value. Then a second time slot started until the situation changed again (from ignorant present to ignorant absent or vice-versa) etc. The number of time slots per individual per experiment varied from 1 to 6 (mean ± SE 1.33 ± 0.06). In total, 233 time slots were generated: 68 in bonobos and 165 in chimpanzees. Of these, 46 time slots occurred where no ignorant individual was present in the party (18 in bonobos and 28 in chimpanzees). For each time slot we compiled the average number of other individuals who had seen the snake present in the party (hereafter potential signalers) weighted for the duration. For example, if one potential signaler was present for 200 seconds and then 4 potential signalers were present for 100 seconds, the average number of other potential signalers was calculated as follows:

$$\frac{1\text{ potential signaller} * 200\text{ sec} + 4\text{ potential signallers} * 100\text{ sec}}{300\text{ (total time in seconds)}} = 2$$

Full null model comparisons and assessment of significance

In each model we tested for species difference in the test predictors by comparing the full model to a null model comprising all test and control predictors, all the random effects and random slopes but without the interaction between species and the test predictors. We tested each full model against its corresponding null model using a likelihood ratio test (LRT, [39]). We then

assessed the significance of each predictor variable using a LRT between the full model and a reduced model comprising all the variables except the one to evaluate. This process was repeated across all variables using the *drop1* function.

Model assumption cheking

For each model we tested for collinearity issue between our predictor variables using the function *vif* from the package "car". Collinearity was not an issue (all vif<2.9). We also assessed model stability removing one level of each random effect at a time and recalculating the estimates of the different predictors, which revealed no stability issue. Finally, we tested for over dispersion in the Model 2b (Poisson error structure), which was not an issue (dispersion parameter=0.83).

SI Results

Calling behavior and time around the snake

The general probability to produce at least one alarm call for any individual within a given experiment day was very similar in both species (median percentage of individuals who saw the snake and also called = 41.7% in chimpanzees and 40.0% in bonobos, **Table S3**). In addition, most individuals produced very few calls during the experiment (75.0% produced less than 12 calls per experiment) but chimpanzees produced in general more calls per experiment (median = 2.9 calls per individual per experiment in chimpanzees and 1.3 in bonobos). The median size of the party around the snake in each experiment was larger in chimpanzees than in bonobos (8 in

chimpanzees and 5 in bonobos) and more individuals saw the snake in each experiment in chimpanzees than in bonobos (median = 4 and 2 respectively). Individuals from both species approached to a similar distance to the snake (median minimum distance to the snake = 2.0 m in both species, **Table S3**).Across all experiments, only one individual produced alarm calls before seeing the snake. After hearing alarm calls from conspecifics, a female chimpanzee came down from a feeding tree and could not locate the snake and gave a series of loud alarm barks alternating looks between the ground (in the opposite direction of the snake) and above her. After 30 seconds she exited the snake area without locating the snake. We excluded this female from the analysis of that experiment day. The interval for each individual who saw the snake and called between seeing the snake and producing the first alarm call was similar in both species (median = 5.0 seconds in chimpanzees and 5.8 seconds in bonobos) as well as the general within-individual inter-call interval (median= 1.7 in chimpanzee and 1.2 in bonobos). Bonobos tended to stay longer around the snake than chimpanzees (median time around the snake = 74 and 54 sec respectively).

Full-null model comparisons

The full-null model comparison was significant in Model 1a (LRT: $X^2 = 6.95$, df=1, $p = 0.008$) indicating that species influenced the change in probability to startle between the first to see the snake and individuals arriving later at the snake. In Model 1b the full-null model comparison was also significant (LRT: $X^2 = 6.32$, df = 1, $p = 0.012$, **Table** 1, Fig. S4) indicating that chimpanzee late arrivers were more likely to have heard a call before seeing the snake than bonobos arriving late at the snake. In contrast, the full null-model model comparison in Model 1c was not significant (LRT: $X^2 = 0.18$, df=1, $p = 0.675$, **Table** 1, Fig. S4) indicating that the likelihood to

have a conspecific with snake-oriented-body when approaching the snake was not significantly different between chimpanzees and bonobos.

The full-null model comparisons testing for species difference in the parameters influencing individual's likelihood to call (Model 2a) and the number of calls produced (Model 2b) were both significant (LRT in Model 2a: $X^2 = 16.67$, df=5, $p = 0.005$, in Model 2b: $X^2 = 19.05$, df=5, $p = 0.002$). In model 2a, the two way interactions between species and 1) individual sex, 2) ignorant in the party, and 3) number of other potential signalers were all not significant (all $P>0.15$). We therefore reran Model 2a removing all these interactions to assess the effect of the single predictors. Similarly we reran Model 2b removing all non-significant interactions (i.e. interaction between species and 1) Individual Sex, 2) number of other potential signalers, 3) presence of ignorant individuals in the party and 4) order of arrival; all $P>0.12$).

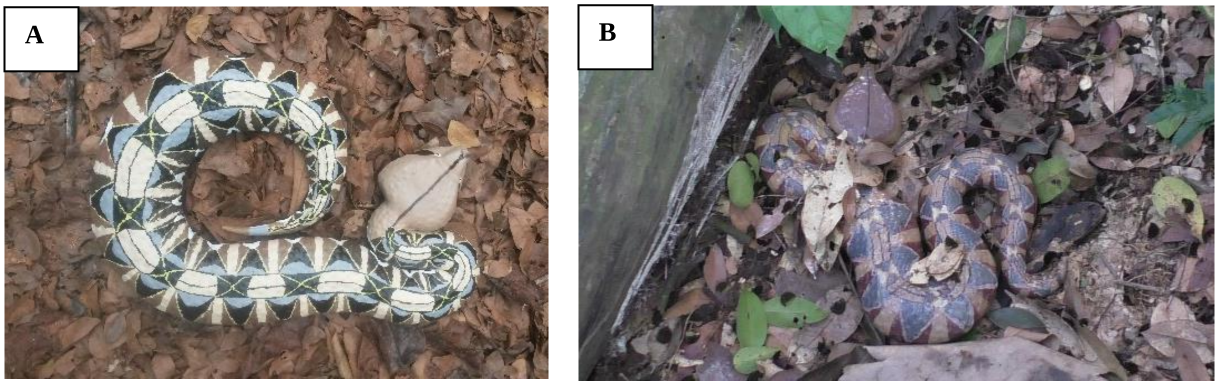

Fig. S1. Example of the 3D printed snake models used for the experiments showing the two different shapes which were designed. B shows how the model was presented: partially covered with vegetation and behind a log.

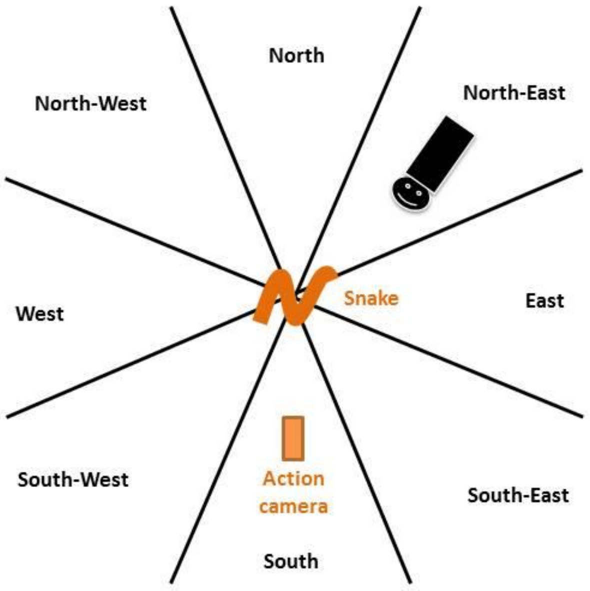

Fig. S2. Schematic representation of the ‘compass’ system used to code the position in space and the body orientation of the apes from the video. The action camera was used as the south direction and all other directions assessed from this stable point. The snake was in the center of the system. The ape (depicted here in black) for example is situated North-East from the snake and facing South-West. The angle between his body orientation and the snake is thus 0 degree and he was considered as facing the snake. This measure was used to assess whether individuals had snake-oriented-body or not when other conspecifics approached the snake.

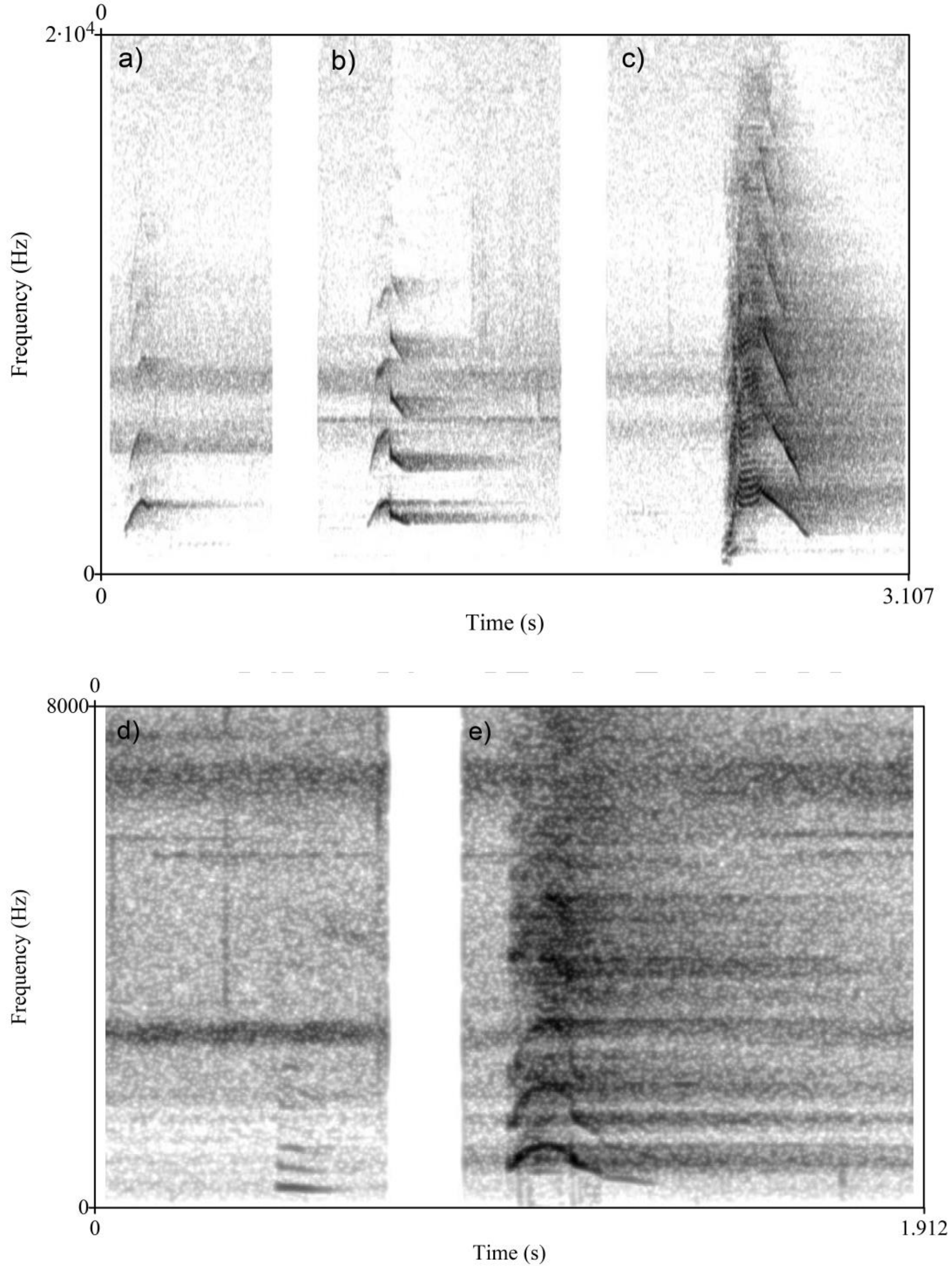

Fig. S3. Spectrogram of the 3 different alarm call types in bonobos: a) soft alarm, b) alarm whistle and c) alarm bark and the 2 alarm call types of the chimpanzees: d) alarm hoo, e) alarm bark. All bonobo calls are from the same adult female and both chimpanzee calls are from the same adult male. Spectrogram settings are as follow; Bonobos: windows length = 0.015 sec, Dynamic range = 60 dB; chimpanzees: windows length = 0.025 sec, Dynamic range = 70 dB.

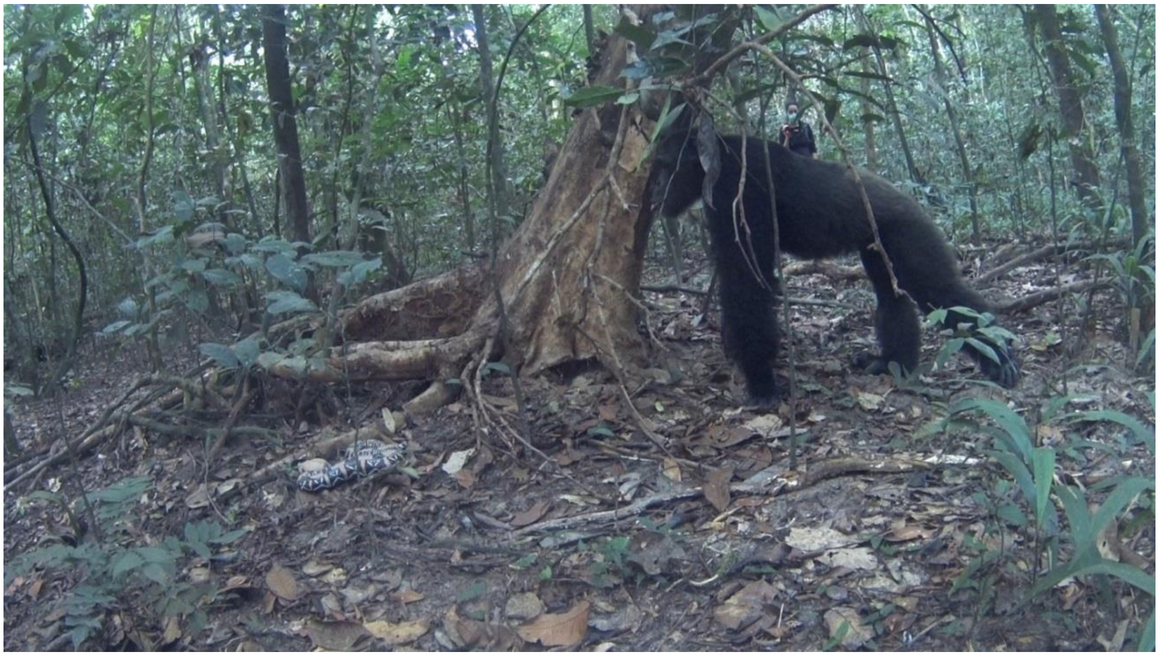

Fig. S4. Example of an individual chimpanzee with snake-oriented-body (i.e. on the ground within 5m of the snake model and with the body oriented toward the snake model).

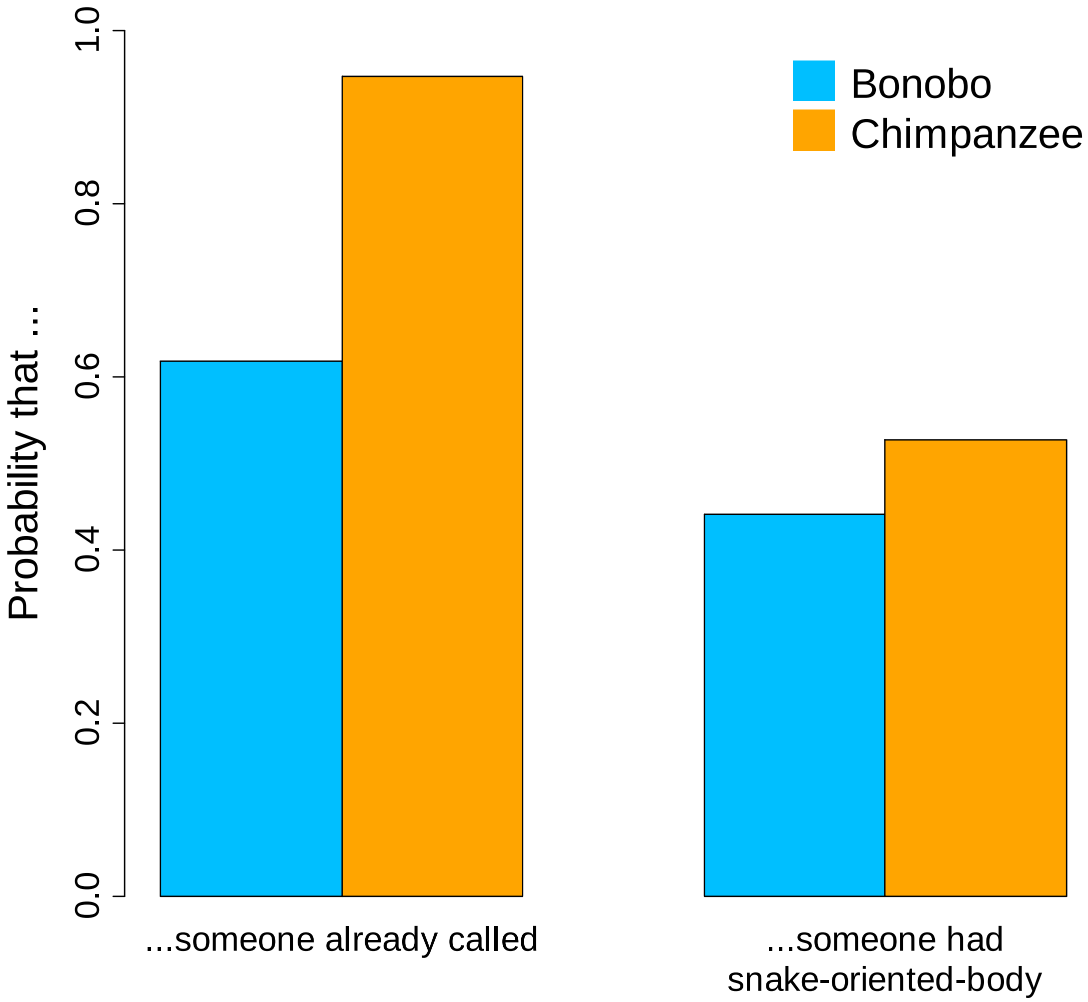

Fig. S5. Information available to late arrivers in bonobos (in blue) and in chimpanzees (orange) (Model 1 b and c). The barplots depict the probability for a late arriver seeing the snake that someone already called (left side) and that someone had snake-oriented-body (left side).

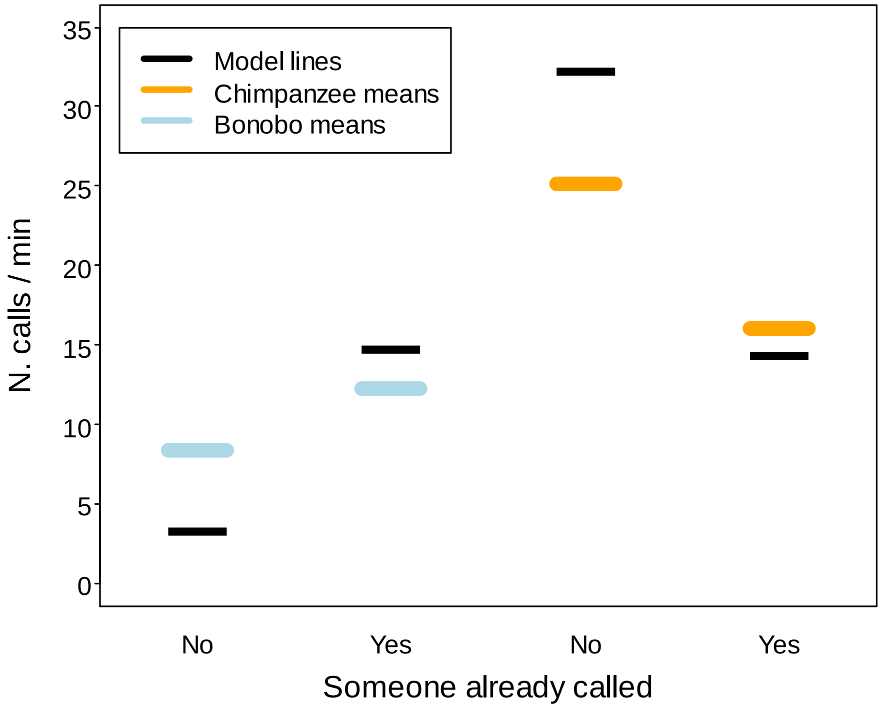

Fig. S6. Number of calls produced by minute in bonobos and chimpanzees when someone already called (right) or not (left; Model 2b). The thick lines represent the mean for bonobos (in blue) and chimpanzees (in orange) for each case. The black lines depict the model lines.

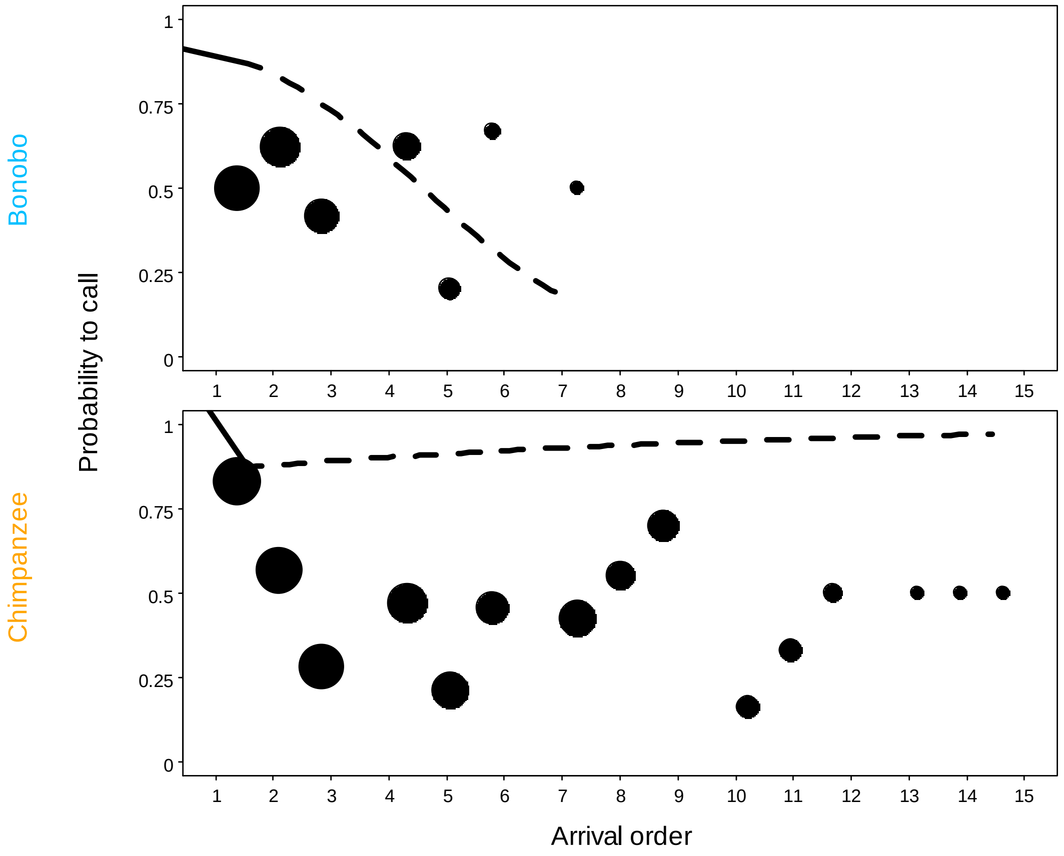

Fig. S7. Probability to call depending on the arrival order at the snake in bonobos (top figure) and chimpanzees (bottom figure) (Model 2a). The black circles represent the data and the area of the circle is proportional to the number of data points in each circle. The dashed line depicts the model line.

Table S1. Composition of the study communities.

		Female		Male		Total
**Study group**	**Species**	*Adult*	*Juvenile*	*Adult*	*Juvenile*	
LK East	Bonobo	10	2	5	4	21
LK Bompusa	Bonobo	16-17	3-5	8	2	29-32
Taï North	Chimpanzee	7	2	4	1	14
Taï South	Chimpanzee	14	4	6	1	25
Taï East	Chimpanzee	9	4	7	4	24

Table S2. Structure of all the final models used in the analysis (after exclusion of non-significant interactions).

	Information transfer efficiency model<br>(Model 1a)	Auditory Information available to late arriver<br>(Model 1b)	Visual Information available to late arriver<br>(Model 1c)	*Triggers of alarm calling*<br>(Model 2a)	*Triggers of the number of alarm calls produced*<br>(Model 2b)
**Response**	Startle upon seeing the snake (Y/N)	Late arriver heard an alarm call before seeing the snake (Y/N)	A conspecific was with snake-oriented-body when the late arriver saw the snake (Y/N)	Alarm called (Y/N)	Number of alarm calls uttered
**Fixed factors**[(1)]	Species<br>First to see the snake (Y/N)<br>*Number of snake seen*<br>*Focal age*<br>*Focal sex*<br>Species : First to see the snake	Species<br>*Focal sex*<br>*Focal age*		Species<br>Heard call (Y/N)<br>Order of arrival<br>Ignorant in the audience (Y/N)<br>Number of knowledgeable individuals<br>*Focal age*<br>*Focal sex*<br>*Number of snake seen*<br>*Time since focal saw the snake*<br>Species : heard a call<br>Species : order of arrival	Species<br>Heard call (Y/N)<br>Order of arrival<br>Ignorant in the audience (Y/N)<br>Number of knowledgeable individuals<br>*Focal age*<br>*Focal sex*<br>*Number of snake seen*<br>Time since focal saw the snake<br>Species : heard a call
**Random Intercepts**	Focal ID<br>Group ID<br>Experiment ID<br>Snake model ID				
**Random Slopes**[(2)]	First to see+ Number of snake seen + Focal sex + Focal age | Group ID<br>Focal sex | Experiment ID<br>Focal age + Number of snake seen | Snake ID	Focal sex + Focal age | Group ID<br>Focal sex + Focal age | Snake ID		Heard call + Ignorant in the audience + Number of knowledgeable individuals + Number of snake seen + Time since focal saw the snake | Group ID<br>Order of arrival + Number of knowledgeable individuals + Number of snake seen |	Heard call + Ignorant in the audience + Number of knowledgeable individuals + Number of snake seen + Time since focal saw the snake | Group ID<br>Order of arrival + Number of knowledgeable individuals |

			experiment ID<br><br>Heard call + Order of arrival + Ignorant in the audience + Number of knowledgeable individuals + Number of snake seen + Time since focal saw the snake | Snake ID	experiment ID<br><br>Heard call + Order of arrival + Ignorant in the audience + Number of knowledgeable individuals + Number of snake seen | Snake ID
**offset**			Log (time slot duration)	

(1): sex was dummy coded with ‘females’ being the reference category; species was dummy coded with ‘bonobo’ being the reference category; age class was dummy coded with ‘adult’ being the reference; and ‘first to see the snake’, ‘heard a call’ and ‘ignorant in the audience’ were all dummy coded with no being the reference category. Control factors are indicated in italic.

(2): for inclusion as random slopes we manually dummy coded and then centred (to a mean of zero) ‘focal sex’, ‘focal age class’, ‘heard a call’ and ‘ignorant in the audience’.

Table S3. Statistical distribution of the social and behavioral parameters related to the snake experiment in both species.

	Chimpanzee					Bonobo				
	median	*min*	*max*	*25%*	*75%*	*median*	*min*	*max*	*25%*	*75%*
Number of exposures to the snake	**2.0**	1.0	7.0	1.0	3.3	**1.5**	1.0	4.0	1.0	2.0
Number of individual present in the party around the snake*	**8.0**	1.0	19.0	5.0	10.5	**5.0**	1.0	15.0	3.5	7.5
Number of individuals who saw the snake per experiment	**4.0**	1.0	12.0	2.5	6.0	**2.0**	1.0	8.0	1.0	3.5
Time spent within 5m of the snake (in seconds)	**54**	1	645	16	165	**74**	7	3337	32	186
Minimum distance of approach (in meters)	**2.0**	0.5	15.0	2.0	4.0	**2.0**	0.5	15.0	2.0	7.5
% of the individuals who see the snake who call (per day)	**41.7**	10.0	100.0	33.3	63.3	**40.0**	0.0	100.0	24.3	52.3
Number of calls per individual (per day)	**2.9**	0.0	206.0	0.5	11.1	**1.3**	0.0	121.0	0.0	11.3
Interval between seeing the snake and first call (when call)	**5.0**	0.3	208.6	2.3	16.2	**5.8**	0.2	433.3	2.3	14.2
Inter-call interval (in seconds)	**1.7**	0.1	672.2	1.3	2.7	**1.2**	0.1	4624.7	0.8	3.1

* The number of individual present in the party around the snake refers to the individuals that the observers could see around the snake (i.e. within50 m). When individuals were alone in the party (i.e. number of individual in the party = 1) they were visually alone but not acoustically alone (there were always other individuals in hearing distance).
In the rare time slots when the individual was alone around the snake (4 in bonobos and 2 in chimpanzees out of 233 slots) we set the factor "presence of ignorant in the audience" to "No" (Model 2a and 2b).

Movie S1: Example of bonobo reaction to the snake model.

An adult female bonobo carrying her infant approaches the snake (placed between the two logs towards the bottom end of the screen in the center). She is the first to approach the snake. At 1 sec on the video she sees the snake and startle and then enters a small tree. At 12 sec on the video but 2min20 sec later in the experiment she moves away from the snake. Importantly she did not produce any alarm call yet despite the fact that other bonobos are present in the party. At 22 sec on the video the female who approached first is still seating on the log but not visible on the video. She still did not utter any call. At 26 sec on the video a juvenile male sees the snake and startles. The other male, who is an adult, also startles. The juvenile then enters a tree and produces a soft alarm call. It is important to note that both individuals did not hear any alarm call before approaching the snake which might be why they startle upon discovering it. At 31 sec on the video, the adult male on the right starts approaching the snake again and the juvenile in the tree resumes alarm calling producing this time an alarm whistle. Both males are brothers.
http://www.youtube.com/watch?v=DAQcEd6P7RU

Movie S2: Example of chimpanzee reaction to the snake model.

An adult male chimpanzee approaches the snake placed behind a root on the left of the screen. Two other adult males and a juvenile orphan female are present in his party. Another party containing the rest of the community is in auditory range of the snake towards the right of the screen. At 12 sec on the video, the male sees the snake but does not startle. He starts producing a series of alarm hoos. At 18 sec on the video (45 sec later in reality) the male starts producing alarm barks. Second 23 on the video he moves towards the back of the snake. At 29 sec on the video (and 2 min 15 sec later in reality) the male stamps with his right hand on the ground in the direction of the snake and produces another alarm hoo. At 37 sec on the video, another adult male, present in the party since the beginning, but who has not seen the snake yet approaches the snake on the right of the screen. At 45 sec on the video, the second male sees the snake and does not startle, possibly because he has heard alarm call before approaching and a conspecific is still present around the snake.
www.youtube.com/watch?v=5t8A_HiTJlc

Additional data table S1 (separate file)

Data used to run Model 1a investigating species differences in the likelihood to startle upon seeing the snake.

Additional data table S2 (separate file)

Data used to run Model 1b investigating species differences in the likelihood to have heard a call before approaching the snake.

Additional data table S3 (separate file)

Data used to run Model 1c investigating species differences in the likelihood have a conspecific with snake- oriented-body when discovering the snake.

Additional data table S4 (separate file)

Data used to run Model 2a investigating species differences in the social factors influencing the likelihood to call.

Additional data table S5 (separate file)

Data used to run Model 2b investigating species differences in the social factors influencing the number of calls produced.
